# Management of Uveitis Patients on Anti-TNF Agents Who Develop Demyelinating Disease – A Case Series

**DOI:** 10.1186/s12348-024-00403-3

**Published:** 2024-07-30

**Authors:** Abel Hamdan, Sumit Sharma, Kimberly Baynes, Rula A. Hajj Ali, Careen Y. Lowder, Sunil K. Srivastava

**Affiliations:** 1 Cole Eye Institute, Cleveland Clinic, 2022 E 105th St I Building, Cleveland, OH 44106 USA; 2 Department of Rheumatology, Cleveland Clinic, Cleveland, OH USA

**Keywords:** Drugs, Immunology, Inflammation, Retina, Treatment medical

## Abstract

**Background/Aims:**

Anti-tumor necrosis factor (Anti-TNF) agents have proven beneficial for the treatment of chronic non-infectious uveitis, yet rare neurological complications and demyelinating disease can occur with their use. Management of uveitis and neurological disease after developing these rare complications is not well understood. We sought to identify these specific cases and their outcomes through a retrospective observational case series.

**Methods:**

Electronic Medical Record (EMR) chart review of 394 non-infectious uveitis patients on anti-TNF therapy focused on identifying patients seen by uveitis specialists at a single institution who were on anti-TNF therapy and had developed neurological symptoms. Cases were reviewed for subsequent management and outcomes of both their neurologic and ocular inflammatory disease.

**Results:**

Five (5) patients were included following complaints of neurological symptoms while on anti-TNF therapy. Subsequent demyelinating diagnosis, acute treatment, and long-term course were described. All five patients continue to be inactive at around three years of anti-TNF discontinuation.

**Conclusion:**

Unidentified rare neurological symptoms and demyelinating disease associated with the use of anti-TNF agents can be detrimental to patient treatment outcomes. Emphasis is given on possible avoidance and early identification of exacerbating underlying disease through a detailed neurologic history and use of imaging when suspicion is high. Patients may have no evidence of higher neurological risk prior to starting an anti-TNF treatment. Discontinuation of an anti-TNF agent and subsequent control of disease is possible with alternative immunosuppressive treatments.

## Introduction

Chronic non-infectious uveitis is a leading cause of vision loss associated with a high risk of ocular complications, including glaucoma, macular edema, and cataracts [1]. Oral corticosteroids and intraocular steroid injections remain a mainstay of treatment for noninfectious uveitis (NIU) despite predictable and serious side effects associated with long-term use [1–4]. Treatment with biologic and other systemic immunomodulatory agents is required in patients using long term corticosteroids greater than the equivalent of 5 mg of prednisone to control their uveitis [1]. Anti-TNF medications, such as adalimumab and infliximab, are modulators which bind to tumor necrosis factor-alpha (TNF-α) and inhibit interaction with surface TNF receptors. Several randomized clinical trials have determined the utility of adalimumab in the treatment of chronic non-infectious uveitis [3, 5, 6]. Rare side effects reported with the use of anti-TNF medications include demyelinating central nervous system (CNS) disorders secondary to use either as a result of the agents themselves or activation of underlying demyelinating disease [1, 7–15]. Immunosuppressive management and long term outcomes of uveitis patients who develop these complications are not clear. In this case series, we will discuss five uveitic patients who presented with neurological symptoms while on anti-TNF agents and their subsequent management.

## Materials and methods

Complying with the tenets of the Declaration of Helsinki, a retrospective institutional review board (IRB) approved case series was created. Study data were collected through EMR chart review of 394 non-infectious uveitis patients on anti-TNF therapy focused on identifying patients seen by uveitis specialists (S.K.S., S.S., and C.Y.L.) at the Cole Eye Institute who were on anti-TNF therapy and had developed neurological symptoms. Patients were excluded if they did not carry a diagnosis of uveitis. Charts were reviewed for demographic information, duration of uveitis, prior immunosuppressive medications, time and type of anti-TNF introduction, initial presentation of neurological symptoms, anti-TNF discontinuation, imaging and labs related to neurological workup, and management following discontinuation. All visits prior to and after anti-TNF introduction were reviewed for evidence of ocular inflammation on clinical exam and imaging. The clinical courses post development of neurological complications were reviewed for outcomes. The time and frequency of uveitis flares along with the use of alternative immunosuppressive treatments were recorded and reviewed. Uveitis flares were defined as new or worsening inflammatory activity on clinical examination or ocular imaging. Descriptive statistics were reported, including mean and range for appropriate variables. We used the CARE checklist when writing our report [16].

## Results

Five (5) patients (1 male; 4 female) with follow-up of four years (2–7) had neurological symptoms while on an anti-TNF agent out of the 394 non-infectious uveitis patients who were on an anti-TNF agent. Average duration of uveitis prior to start of anti-TNF agents was two years (4 months-5 years). Mean number of immunosuppressive medications prior to starting anti-TNF agents was three. Average number of months from anti-TNF initiation to neurological symptoms and discontinuation was nine (4–20) and 14 (4–36), respectively. Two of five patients were positive for oligoclonal antibodies. All five patients had hyperintense lesions on brain MRI. Mean number and time of uveitic flares three years following discontinuation were two (0–3 flares) most commonly around 20 months (1 month–3 years), respectively. All five patients continue to be inactive after at least two years follow-up. The case scenarios of each of the five patients are briefly summarized below:

### Case 1

A female patient with a past medical history (PMH) of chronic panuveitis OD, iritis OS, and migraines on adalimumab (Humira™, Abbvie, Chicago, IL) for four months endorsed intermittent mid-sternal chest pain and episodic right groin/hip pain for a week followed by new onset right-sided weakness. She had three years of previous unsuccessful treatments with prednisone, mycophenolate, methotrexate, topical prednisolone acetate drops and two intraocular steroid injections. Patient was sent to the emergency department (ED); cardiac work-up was negative and she was discharged home. She immediately followed up with Ophthalmology; review of systems was positive for blurred vision and photophobia in the right eye. Visual Acuity (VA) was 20/25 OD and 20/20 OS, her intraocular pressure was normal, she had a + 2 Afferent Pupillary Defect (APD) OD and had full ocular ductions. Slit lamp and dilated fundus examination were unremarkable. Optical Coherence Tomography (OCT) and Fluorescein Angiography (FA) imaging were stable. Her visual field had worsened with circumferential constriction in the right eye. Baseline MRI brain and orbits w/wo six months prior was unremarkable; MRI brain and orbits w/wo on day of neurological presentation showed a new right dorsolateral C-spine lesion w/ faint punctate enhancement on dorsal border. She was admitted and received a three day course of intravenous methylprednisone 1 g with partial improvement of her paresthesia. Inpatient MRI of cervical spine showed a right medullary demyelinating plaque. Neuromyelitis optica (NMO) and myelin oligodendrocyte glycoprotein (MOG) antibodies were negative. Her cerebrospinal fluid (CSF) studies revealed oligoclonal bands and she was diagnosed with multiple sclerosis (MS). The patient was started on rituximab (Ruxience™; Pfizer, New York, NY) 1000 mg in NaCl 0.9% 500mL every six months, yet developed a worsening ocular flare within a month while on prednisolone drops QOD; she required additional local steroid control, including a dexamethasone 0.7 mg implant (Ozurdex™, Abbvie, Chicago, IL) followed by a surgical fluocinolone acetonide 0.59 mg implant (Retisert™, Bausch and Lomb, Laval, Canada). MRI imaging three months following rituximab initiation showed stable brain MRI; cervical MRI two years later showed three new lesions which have since been stable. She endorsed a new ocular flare and worsening right hand weakness following three years of inactivity. Her flare was controlled using an additional intraocular dexamethasone 0.7 mg implant and surgical fluocinolone acetonide 0.59 mg implant replacement. Her neurology consult in the months following revealed no new remarkable findings; she continues to tolerate rituximab well.

### Case 2

A female patient with PMH of pars planitis and retinal vasculitis on mycophenolate mofetil (MMF) 2 g daily for six months presented with bilateral blurred vision. After continued inflammation despite increasing the MMF dose to 3 g, adalimumab (Humira™, Abbvie, Chicago, IL) was added with major improvement in imaging and her symptoms. Three months later, she developed acute onset visual changes in the right eye, described as a “gray cloud” in her visual field and color desaturation. There was no pain with eye movements. VA 20/50 OD and 20/40 OS, APD OD, and Ishihara test scored 20/27 OD and 27/27 OS. Demyelinating disease was suspected; MRI brain and orbits revealed enhancement of the right optic nerve. No other acute intracranial findings or evidence of demyelinating disease were found. NMO and MOG antibodies were negative. There was no record of lumbar puncture (LP). Adalimumab was discontinued; she was admitted and treated with methylprednisolone 1 g daily for three days. Further exploration of her PMH revealed she had prior episodes of numbness in her right leg, left arm, and intermittent jerking years prior. She was discharged with an oral prednisone 60 mg taper and restarted mycophenolate 3 g daily. At two years neurological follow-up, her MRI brain has remained stable; she has been inactive without additional neurologic or ophthalmic flares.

### Case 3

A female patient with PMH of pan-uveitis secondary to Vogt-Koyanagi-Harada presented six months after initiation of adalimumab (Humira™, Abbvie, Chicago, IL) with left eye pain, photopsias, and blurry vision. Her Vogt-Koyanagi-Harada was initially treated with steroids then escalated to adalimumab with resolution. Her visual acuity was 20/20 OD and OS VA 20/40 (previously 20/25 a week ago) and she had 40% red desaturation in her left eye. Left APD and pain with left eye movements was noted. MRI brain was remarkable for multiple T2 hyperintensities. LP findings were significant for elevated immunoglobulin G (IgG) index and positive oligoclonal bands. NMO antibodies were negative; there was no record of a MOG antibody study. Neurology consult noted previous episodes of double vision and episodic paresthesias in her hands since starting adalimumab. Adalimumab was held; she was admitted and given a three day course of intravenous methylprednisone 1 g with partial improvement. The patient was started on ocrelizumab (Ocrevus™, Genentech USA, San Francisco, CA) with good tolerance. The patient had additional flares and worsening MRI lesions in the two years following discontinuation of adalimumab. At three year follow-up she was inactive with no additional ophthalmic or neurological flares; her most recent MRI at three years showed no new T2 or enhancing lesions.

### Case 4

A female patient with biopsy-proven sarcoidosis was started on infliximab (Remicade™, Janssen Pharmaceuticals, Beerse, Belgium) 500 mg (5 mg/kg) every 6 weeks and methotrexate 25 mg weekly for bilateral anterior uveitis and systemic disease. Infliximab was continued for three years until she endorsed intermittent right-sided weakness, numbness, and tingling progressing over the last two years. Other nonspecific complaints included short-term memory problems, blurred vision, and headaches. Her right-sided symptoms progressed to constant weakness primarily affecting her right side. Eye examination at an outside facility was unremarkable. Prior electromyography (EMG) was unremarkable. MRI at neurological symptom presentation showed a discrete hyperintense T2 lesion in the posterior cervical cord and multiple white matter lesions in the brain. Previous MRI three years prior was unremarkable. NMO and MOG antibodies were negative. LP findings were negative for oligoclonal bands. Infliximab was held; no treatment was given at this time, however an exacerbation of uveitis one month later required methylprednisolone 1 g rescue. Rituximab (Rituxan™, Genentech USA, San Francisco, CA) was initiated with methotrexate 25 mg subcutaneous weekly. Her uveitis flared once in the month following infliximab discontinuation, yet resolved with continuation of the rituximab and methotrexate. Repeat MRIs have been stable since stopping infliximab.

### Case 5

A male patient with PMH of psoriatic arthritis, bilateral panuveitis and retinal vasculitis presented endorsing a year-long history of worsening leg and back spasms. At the time he had been taking methotrexate 25 mg and adalimumab (Humira™, Abbvie, Chicago, IL) for two years. VA 20/20 OU with ocular examination showing no signs of inflammation. MRI spine and brain revealed demyelinating lesions in the thoracic spine and cortex. Neurologic evaluation did not reveal any additional symptoms or clinical findings. There was no record of lumbar puncture, NMO, or MOG studies. Adalimumab was discontinued without acute treatment due to chronicity, and the patient was already on oral steroids. Alternative treatment with rituximab (Rituxan™, Genentech USA, San Francisco, CA) and later secukinumab (Cosentyx™, Novartis, Basel, Switzerland) were initiated, both of which failed due to worsening MS symptoms and enlarging lesions, respectively. Ustekinumab (Stelara™, Janssen Pharmaceuticals, Beerse, Belgium) with methotrexate 25 mg was initiated with good tolerance and no new symptoms. The patient had two uveitis flares within three years following discontinuation of adalimumab; he was inactive at his seven year follow-up. Repeat MRI at three years following discontinuation showed one new T2 lesion that has since been stable at five year follow-up.

## Discussion

TNF-a is produced initially as a transmembrane molecule (tmTNF) and is subsequently released from the cell as a soluble cytokine (solTNF) via regulated cleavage of tmTNF by TNF-α converting enzyme (TACE). Both forms of TNF are biologically active and interact with two distinct receptors—TNFR1 and TNFR2. Studies suggest a dichotomy between solTNF and tmTNF, in which multiple sclerosis is associated with the detrimental effects of solTNF via TNFR1, but tmTNF signaling via TNFR2 is important for repair and remyelination [17] Introduction of more selective anti-TNF agents or more efficient dose adjustment may hold the key to better outcomes [18, 19]. As current anti-TNF agents affect both pathways, the blocking of the protective features of TNFR2 and the remyelinating process could potentially lead to demyelinating events. Therefore, selective TNF inhibition or activation of TNFR2 could lead to a new treatment approach for inflammatory disease. Several theories have been proposed in an attempt to clarify the potential biological role of TNF-α blockers in triggering or aggravating demyelination such as increasing CNS TNF-a levels, decreasing TNFR2 receptors, or altering other inflammatory mechanisms [20, 21].

Five TNF-α modulators are currently approved for clinical use (etanercept, infliximab, adalimumab, golimumab, and certolizumab) [20]. Etanercept in a receptor blocker, while the latter are direct antibody blockers [20]. A variety of manifestations of demyelinating disorders are associated with anti-TNF-α therapy and include, in descending order, optic neuritis, multiple sclerosis, demyelinating neuropathy, Guillain-Barre syndrome (GBS), and transverse myelitis, all of which have a fairly low incidence [22].

Demyelinating complications related to anti-TNF have been shown to be exceedingly rare across a number of different studies. CNS demyelinating diseases were diagnosed in 13 out of 39,933 (0.03%) patients exposed to biologics included in five studies across different specialties [23]. The incidence rates of serious demyelinating disorders were ≤ 0.1 events/100 person-years (PYs) in a review of 23,458 patients exposed to adalimumab in 71 global clinical trials in rheumatoid arthritis (RA), juvenile idiopathic arthritis (JIA), ankylosing spondylitis (AS), psoriatic arthritis (PA), psoriasis (Ps) and Crohn’s disease (CD) [24]. CNS demyelinating diseases were also noted in 35 of about 13,500 (0.26%) rheumatology patients [25]. The BIOGEAS registry contains nearly 13,000 autoimmune disease cases developed after patients were exposed to biologics; of these cases, 803 were specific for CNS demyelinating diseases (6%). Specific characteristics of induced demyelinating diseases were available in 651 cases, of whom 254 were classified as multiple sclerosis (MS)/MS-like and 523 as neuromyelitis optica (NMO) [23]. Underlying diseases were detailed in only 184 of these cases, yet the most frequent were rheumatoid arthritis/juvenile idiopathic arthritis (RA/JIA) in 66 (36%) and inflammatory bowel disease (IBD) in 66 (36%). About 80% of biologics administered in BIOGEAS consisted of etanercept and infliximab [23].

Higher risk for demyelinating events based on current literature includes patients on anti-TNF agents (most reported include etanercept, infliximab, and adalimumab) and patients who have a history of RA/JIA or IBD, although this is still somewhat controversial [23, 26]. The association of inflammatory CNS events with TNF inhibitor exposure was observed in patients with rheumatoid arthritis. No association was observed in the remaining pooled autoimmune diseases (ankylosing spondylitis, psoriasis and psoriatic arthritis, Crohn disease) suggesting differences in association depending on underlying autoimmune disease, yet this is still unclear [26].

Kemanetzoglou et al. 2017 reviewed 122 cases of CNS demyelination associated with TNF-α blockers published in the medical literature between January 1990 and August 2016. Neurological recovery varied; follow-up of cases after stopping anti-TNF agents during this time revealed complete recovery in 44 patients (36%), partial in 26 patients (21%), whereas no resolution of symptoms was described in 34 patients (28%) [20]. Additional articles continue to be published correlating anti-TNF agents with neurological disease [27–35].

Anti-TNF medications were specifically tested for ocular treatments and side effects through the VISUAL randomized controlled trials (RCTs) [5, 6]. 1 of 111 uveitis patients in the VISUAL I multinational phase 3 RCT for adalimumab had a serious adverse event related to demyelination (1.6 events/100 patient years), yet none were reported in the VISUAL II RCT [5, 6]. The incidence of demyelinating disorders reported in the VISUAL III study (6 of 424 uveitis patients) (0.5 events/100 patient years) was higher than that observed in clinical trials for other adalimumab indications (< 0.1 events/100 patient years), yet the observed rate was comparable to the background rate reported in patients with uveitis who were not exposed to adalimumab [3]. Similarly, optic neuritis and demyelinating peripheral nervous system disease was reported in association with the use of other anti-TNF agents [5, 36–38]. Although CNS demyelination after treatment with TNF-α blockers is not necessarily associated with the duration of the therapy and drug discontinuation does not always lead to improvement, treatment should be discontinued at the appearance of unexplained neurologic symptoms.

Acute management of patients presenting with neurological symptoms while on an anti-TNF agent typically follows the treatment recommendations for acute exacerbations of multiple sclerosis, including intravenous (IV) or oral corticosteroids, with good results in the short term, although the long-term course of the demyelinating disease appears unpredictable [38, 39]. Currently, there is very limited literature describing successful alternative long-term management specific to uveitis patients presenting with neurological symptoms while being on an anti-TNF agent. A total of seven other detailed cases were identified reporting induced demyelination related to the use of anti-TNF medications specifically for uveitis [35, 40–43]. Our experiences are noted in Table 1. Additional demyelination cases were noted in other previous uveitis studies, yet details from those specific cases were limited and not included in the figures. [3, 5, 44, 45].

**Table 1:** Cases of non-infectious uveitis patients on anti-TNF agents who presented with neurological symptoms and were reported as induced demyelination.

Sex	Disease Indication For Anti-TNF Agent	Anti-TNF Agent Used	Neurological Symptoms	Immediate Rescue Treatment	Alternative Long-Term Treatments	Presumed Native MS	Oligoclonal Bands	NMO Antibodies	MOG Antibodies	Normal Baseline MRI
Female	Panuveitis	Adalimumab	Chest/Groin Pain; Vision Loss	IVMP	Rituximab; Intraocular Steroid Implants	Yes	Yes	No	No	Yes
Female	Pars Planitis/Retinal Vasculitis	Adalimumab	Vision Loss	IVMP	Mycophenolate	No	N/A	No	No	N/A
Female	Panuveitis	Adalimumab	Vision Loss/Eye Pain	IVMP	Ocrelizumab	Yes	Yes	No	N/A	N/A
Female	Anterior Uveitis	Infliximab	Hemibody Anesthesia	IVMP	Rituximab; Methotrexate	No	No	No	No	Yes
Male	Panuveitis / Retinal Vasculitis	Adalimumab	Leg/Back Spasms	Oral Steroids	Ustekinumab; Methotrexate	Yes	N/A	N/A	N/A	N/A

Anti-TNF: Anti-Tumor Necrosis Factor, IVMP: Intravenous methylprednisolone, MS: Multiple Sclerosis, NMO: Neuromyelitis Optica, MOG: Myelin Oligodendrocyte Glycoprotein, N/A: Not Available

Still a controversial question, “Should patients always receive an MRI prior to starting anti-TNF therapy for autoimmune disease?” Kaltsonoudis et al., 2014 exemplified possible benefits by identifying patients to exclude from treatment. 77 patients eligible for anti-TNFα therapy were evaluated with MRIs prior to start, at 18 months, or after developing neurological symptoms. Two patients in this study failed prescreening and did not receive anti-TNFα therapy because brain MRIs at baseline revealed lesions compatible with demyelinating diseases. Three additional patients also developed neurological symptoms (optic neuritis, facial paresis, and peripheral neuropathy) later in the study at 6, 8, and 25 months. Only the patient at 8 months had demyelinating lesions on follow-up MRI [46]. Did treatment of these patients activate latent MS? Similar to our and others’ cases, it is difficult to be sure as patients may have no abnormalities on MRI prescreening [20]. Most available information on ocular adverse events associated with anti-TNF-α therapy comes from uncontrolled studies, therefore solid conclusions cannot be drawn [47]. This reemphasizes the need to clearly address patient safety screening and monitoring when considering immunomodulatory therapy and to utilize the input of multiple medical specialties, as communication among healthcare professionals fosters optimal diagnosis and therapy [1, 15]. Taylor et al., 2021 highlighted drawbacks to increased use of MRIs. Specifically evaluating the risk of demyelination is highly complex, with little data to inform risk-benefit discussions. Performing screening MRI on all patients is also not feasible from a resource perspective and confers a risk of significant anxiety in patients as a result of incidental diagnoses with varying degrees of clinical significance being revealed [25].

Our series does have some limitations. The retrospective nature of our series prevents us from determining an overall complication rate. The presence of previous immunosuppressive use in all of these cases could confound the association between anti-TNF and demyelination complications. The low incidence of demyelination overall does make it difficult to make broad conclusions based on these cases. However, given the rarity of this complication, our series is the largest to date in uveitis patients. We presented five interdisciplinary cases in which we managed patients with adverse neurological reactions while on an anti-TNF agent. When symptoms related to neurological disease were identified, anti-TNF treatment was stopped, patients had appropriate MRI/steroid management, and immune suppressive medications were changed to alternative long-term medications. These included anti-metabolites, alternative biologics (rituximab) and local steroid implants when needed. Following their acute episodes, our patients have been overall stable with partial or complete resolution of their symptoms; all but one continues without flares or progression of symptoms. Within our experiences and with these limitations in mind, we conclude that patients who present with adverse neurological reactions while on anti-TNF agents can be rescued while minimizing negative sequelae when given appropriate MRI/steroid management and alternative long-term medications.

### Financial disclosures

Sumit Sharma - Cole Eye Institute, Cleveland Clinic.

- Grants/Contracts.
  - Allergan - To Institution.
  - Genentech/Roche - To Institution.
  - Gilead - To Institution.
  - IONIS - To Institution.
  - Santen - To Institution.
- Consulting Fees.
  - Abbvie - Personal.
  - Genentech/Roche - Personal.
  - Regeneron - Personal.
  - Clearside - Personal.
  - Bausch and Lomb - Personal.
  - Eyepoint - Personal.
  - RegenxBio - Personal.

Rula A. Hajj Ali - Department of Rheumatology, Cleveland Clinic.

- Royalties/Licenses.
  - UpToDate - Personal.

Sunil K Srivastava - Cole Eye Institute, Cleveland Clinic.

- Grants/Contracts.
  - Allergan - To Institution.
  - Eyepoint - To Institution.
  - Gilead - To Institution.
  - Regeneron - To Institution.
  - Santen - To Institution.
- Consulting Fees.
  - Abbvie - Personal.
  - Adverum - Personal.
  - Regeneron - Personal.
  - jCyte - Personal.
  - Bausch and Lomb - Personal.
  - Eyepoint - Personal.
  - RegenxBio - Personal.

## Data Availability

All topic-relevant, deidentified data analyzed are included in this article.

## Declarations

### What is already known on this topic

Rare side effects associated with anti-TNF medications include rare neurological complications and demyelinating disease. Immunosuppressive management and long term outcomes of uveitis patients who develop these complications are not clear.

### What this study adds

Patients may have no evidence of higher neurological risk prior to starting an anti-TNF treatment. Discontinuation of an anti-TNF agent and subsequent control of disease is possible with alternative immunosuppressive treatments.

### How this study might affect research, practice or policy

Despite the absence of any prior risk factors, practitioners should be cautious of new neurological disease in patients who present with neurological symptoms while on an anti-TNF medication. Patients who present with adverse neurological reactions while on anti-TNF agents can be rescued while minimizing negative sequelae when given appropriate magnetic resonance imaging (MRI)/steroid management and alternative long-term medications.

### Synopsis/Precis

Patients may have no evidence of higher neurological risk prior to starting an anti-TNF treatment. Discontinuation of an anti-TNF agent and subsequent control of disease is possible with alternative immunosuppressive treatments.
